# TSA-HGNN: a stability-aware multi-scale temporal graph neural network for dynamic community detection

**DOI:** 10.3389/frai.2026.1824901

**Published:** 2026-05-28

**Authors:** Gowthami Vusirikkayala, V. Madhu Viswanatham

**Affiliations:** School of Computer Science and Engineering, Vellore Institute of Technology, Vellore, India

**Keywords:** dynamic community detection, echo state network, evolving networks, GraphSAGE, informer, temporal modeling, temporal smoothness

## Abstract

Dynamic community detection is an increasingly important research topic in network science. In real-world networks, edges and nodes change over time; therefore, the community structure must evolve. Due to the dynamic nature of networks, many community detection algorithms rely on static graph assumptions and may not perform well in dynamic networks. Furthermore, the community assignments between snapshot graphs may not be stable. In this study, we designed a stability-aware, multi-scale temporal graph neural network, TSA-HGNN, for dynamic community detection. The proposed TSA-HGNN framework comprises three main components. First, we utilized GraphSAGE to obtain the snapshot-level spatial embedding. Second, the GraphSAGE outputs are fed into two temporal components to model multi-scale temporal patterns in evolving graphs: a temporal convolutional network (TCN) for short-term patterns and an Informer with ProbSparse attention for long-term patterns. Third, we adopted an echo state network (ESN) as an auxiliary component. Moreover, we imposed an additional temporal smoothness constraint on the output community assignments to further enhance stability across consecutive snapshots. To facilitate stable training, we propose a joint optimization framework for TSA-HGNN. Experimental results on three main benchmark datasets (Community Detection Dataset, Reddit Hyperlink Network, and DBLP Collaboration Network), together with an additional temporal benchmark dataset for extended evaluation, validate the superior performance of TSA-HGNN compared with state-of-the-art algorithms DeepWalk, Node2Vec, TGN, TGAT, and GCN-LSTM. The accuracy values for these datasets are 0.9843, 0.9755, and 0.9931, respectively. The F-Score, Modularity Q, NMI, and ARI for TSA-HGNN are 0.9807, 0.8273, 0.8878, and 0.8677, respectively, and achieve state-of-the-art results. Furthermore, temporal stability is further supported by explicit community switch-rate analysis, and the reported improvements are reinforced by corrected statistical significance tests across the main evaluation metrics. Therefore, TSA-HGNN not only enables dynamic community detection more efficiently but also achieves highly stable results.

## 1 Introduction

Networked data are now central to many real-world systems, including social and communication platforms, biological interaction networks, transportation systems, and scientific collaboration graphs. In these settings, the underlying graph is rarely static. Nodes and edges appear, disappear, and reorganize over time, and the community structure evolves accordingly. Therefore, community detection becomes a temporal problem rather than a purely structural one, especially when the goal is to capture meaningful group evolution without introducing unstable or artificial community transitions (Wang et al., 2023; Qiu et al., 2022; Huang and Gu, 2023; Pan et al., 2024).

Dynamic community detection remains important in applications such as social mining, biological pathway analysis, cybersecurity, recommendation systems, and temporal scientific network analysis (Ranjkesh et al., 2024; Yuan et al., 2025). Compared with static community detection, the dynamic setting is substantially more challenging for three reasons. First, the graph structure changes over time, so a single snapshot cannot adequately represent the system. Second, temporal dependencies may operate at different scales: some community changes are local and short-lived, whereas others emerge gradually over longer horizons. Third, community assignments should remain stable across adjacent snapshots unless the observed network evolution provides strong evidence of genuine structural change. A practical dynamic community detection model must therefore balance structural quality, temporal adaptivity, and assignment stability.

Existing approaches to dynamic community detection generally fall into three broad categories. The first applies a static method independently to each snapshot, then links the resulting partitions over time. The second updates communities incrementally as nodes and edges change, usually by optimizing a temporal trade-off objective. The third model jointly captures multiple snapshots to capture longer-range temporal dependencies. Although each direction has produced useful results, none is universally satisfactory. Snapshot-wise methods often ignore temporal continuity, incremental methods can be sensitive to update order and parameter settings, and joint temporal methods may still struggle to preserve stable community evolution while remaining computationally efficient (Rossi et al., 2020; Sankar et al., 2020; Pareja et al., 2020).

In parallel, graph representation learning has improved the quality of node embeddings for downstream clustering and community analysis. Inductive graph neural models such as GraphSAGE are particularly attractive in evolving settings because they can generate node representations from neighborhood information without relying on a fixed transductive embedding table (Hamilton et al., 2017). However, snapshot-level graph encoders alone do not capture temporal dynamics. Sequence models such as temporal convolutional networks (TCNs), Informer-style sparse attention, and reservoir-based recurrent systems are well-suited for modeling temporal signals, but they do not by themselves account for graph structure at each time step (Bai et al., 2018; Zhou et al., 2021; Jaeger, 2001). This creates a clear methodological gap: dynamic community detection requires a framework that can jointly encode snapshot topology, short-range temporal changes, long-range temporal dependencies, and stable community evolution.

To address this gap, we propose TSA-HGNN, a Temporal Stability-Aware Hybrid Graph Neural Network for dynamic community detection. TSA-HGNN integrates four complementary components into a unified pipeline. GraphSAGE is used to obtain inductive spatial embeddings for each snapshot. A TCN captures short-range temporal evolution and local structural reorganization. An Informer with ProbSparse attention models longer-range dependencies more efficiently than dense temporal attention. An echo state network (ESN) provides lightweight non-linear temporal memory. Furthermore, a temporal smoothness constraint is introduced to reduce abrupt changes in community assignments across adjacent snapshots. The result is a framework designed not only for strong clustering performance but also for temporally consistent and computationally efficient dynamic community discovery.

The major contributions of this work are as follows:

- We propose a multi-scale dynamic community detection framework that combines GraphSAGE for inductive snapshot-level spatial encoding, a TCN for short-range temporal evolution, an Informer with ProbSparse attention for long-horizon dependency modeling, and an ESN reservoir for efficient non-linear temporal memory (Hamilton et al., 2017; Bai et al., 2018; Zhou et al., 2021; Jaeger, 2001).
- We introduce a stability-aware optimization objective that explicitly regularizes variation in snapshot-to-snapshot embeddings, thereby reducing abrupt community transitions while preserving meaningful temporal evolution.
- We design the framework to balance predictive quality and computational efficiency by combining sparse long-range temporal modeling with lightweight reservoir memory, rather than relying entirely on fully trainable recurrent temporal modules.
- We support the proposed framework with a rigorous empirical evaluation across multiple benchmark settings, an explicit analysis of temporal stability, and statistical validation of the principal clustering and structural metrics.

The remainder of the paper is organized as follows. Section 2 reviews related work in dynamic community detection and higher-order approaches. Section 3 presents the proposed TSA-HGNN framework. Section 4 reports the experimental results and analysis. Section 5 concludes the paper and outlines directions for future work.

## 2 Literature review

### 2.1 Higher-order and hypergraph methods

Although the present study focuses on dynamic pairwise graph snapshots, it is important to acknowledge that some real-world interactions are inherently higher-order. Examples include co-authorship groups, group messaging, and collective participation events, where relationships naturally involve more than two entities. Hypergraph-based models are therefore relevant to community detection because they can represent such multi-way interactions more directly than standard pairwise graphs. HyperGCN extends graph convolution to hypergraph structures via a Laplacian-based approximation, while OA2H-SP introduces an adaptive anchor-based hypergraph learning strategy for efficient community discovery (Yadati et al., 2019; Berahmand et al., 2025). These approaches are valuable because they capture higher-order structural dependencies that may be hidden when the data are reduced to ordinary pairwise links.

However, these methods are not directly comparable to the problem addressed in this paper. TSA-HGNN is designed for dynamic community detection on evolving pairwise graph snapshots, explicitly modeling short-range temporal change, long-range temporal dependencies, and snapshot-to-snapshot stability. Conversely, existing hypergraph approaches are primarily formulated for static hypergraph clustering or other learning settings, and they do not explicitly incorporate temporal smoothness constraints between consecutive snapshots. Therefore, hypergraph methods should be viewed as closely related but not strictly one-to-one baselines under the current formulation. They are most useful for clarifying the broader methodological landscape and for identifying future extensions of TSA-HGNN toward temporal hypergraph settings.

### 2.2 Dynamic community detection methods

Dynamic community detection using learning and optimization methods has been widely studied in recent years. Existing approaches include adversarial learning, contrastive cross-snapshot supervision, attention-based temporal graph representation learning, and hybrid methods that combine learned embeddings with explicit partitioning rules. For example, GANSE learns node representations using a semi-supervised generative adversarial network, enforces structural consistency through graph rewiring, and performs reconstruction-guided partitioning with an additional mechanism for isolated nodes (Liu X. et al., 2024). CD2NE-GCN combines Node2Vec-style embeddings with a graph convolutional network to improve clustering quality and compares the resulting partitions against unsupervised graph learning baselines (Bhattacharya et al., 2023). STEC-Net learns snapshot-wise node representations using GCN, models temporal evolution through GRU-based temporal components, and obtains final communities using a self-organizing map (Xu and Chu, 2025).

A related line of work studies community evolution using explicit optimization or evolution-aware mechanisms. De Silva et al. investigated modularity-based graph partitioning and attribute-aware community detection, and proposed an evolution mechanism based on reinforcement learning and birth–death processes (de Silva et al., 2025; Pi et al., 2025). CL-OND introduced a contrastive learning framework for evolving graphs, treating adjacent snapshots as strongly correlated samples and using a non-aligned neighbor contrastive loss to capture temporal patterns across snapshots (Li et al., 2025). Furthermore, a propagation-attraction strategy has been proposed to identify community backbones and refine final partitions through attraction rules (Fu et al., 2025). These methods show that temporal dependency is central to dynamic community discovery, but they also highlight the ongoing difficulty of balancing structural quality, temporal adaptivity, and stability.

### 2.3 Directed, parameter-sensitive, and stability-oriented models

Several recent studies have examined issues particularly relevant to realistic dynamic graphs, including directionality, parameter sensitivity, and robustness to perturbations. In directed networks, Yu et al. (2025) proposed direction-aware node embeddings based on the regularized Laplacian and used collaborative clustering with Gray Wolf Optimization to adaptively estimate key clustering parameters across different directed connectivity modes. Wang et al. designed a dynamic graph model that leverages temporal semantics from earlier graph snapshots and topic-shift signals to improve graph representation learning on real-world data (Wang et al., 2023). Qu and Ma proposed a Fourier–fractional neural framework with Lyapunov-style stability arguments to reduce oscillations under perturbation, thereby emphasizing the broader importance of stable temporal community analysis (Qu and Ma, 2026). Other hybrid methods based on evolutionary search, attention mechanisms, and modularity optimization have also been reported, showing the diversity of current approaches to robust community discovery (Zhu et al., 2023; Tang et al., 2025; Liu B. et al., 2024; Al-Andoli et al., 2024; Zhang et al., 2026).

### 2.4 Dynamic GNNs and temporal sequence models

Recent dynamic graph neural networks such as TGN, DySAT, and EvolveGCN have improved temporal node representation learning by introducing event-driven memory, temporal self-attention, or time-evolving graph convolutional parameters (Rossi et al., 2020; Sankar et al., 2020; Pareja et al., 2020). These models have demonstrated strong performance in dynamic graph representation learning, but they also exhibit several limitations for dynamic community detection. In particular, they do not always model long-horizon temporal dependencies efficiently; they can become computationally expensive on large, evolving graphs; and they typically lack an explicit stability mechanism to prevent abrupt changes in communities between adjacent snapshots.

Sequence models offer another useful perspective. TCN captures temporal dependencies through causal dilated convolutions and is effective for short-range temporal evolution (Bai et al., 2018). Informer reduces the cost of long-sequence attention through ProbSparse selection while preserving long-range temporal modeling capability (Zhou et al., 2021). ESN provides non-linear temporal memory with low training overhead because only the readout layer is optimized (Jaeger, 2001). However, these sequence models are not graph encoders on their own and do not directly incorporate the snapshot topology. This motivates a hybrid design in which snapshot-level graph structure is first encoded and then modeled through complementary temporal modules.

Overall, the literature indicates that dynamic community detection still lacks a unified framework that simultaneously provides inductive spatial encoding, short-range temporal adaptation, long-range dependency modeling, and explicit stability control across snapshots. This gap motivates the proposed TSA-HGNN framework, which combines GraphSAGE, TCN, Informer, ESN, and stability-aware optimization into a single pipeline for dynamic community detection.

## 3 Proposed methodology

**Figure 1:**
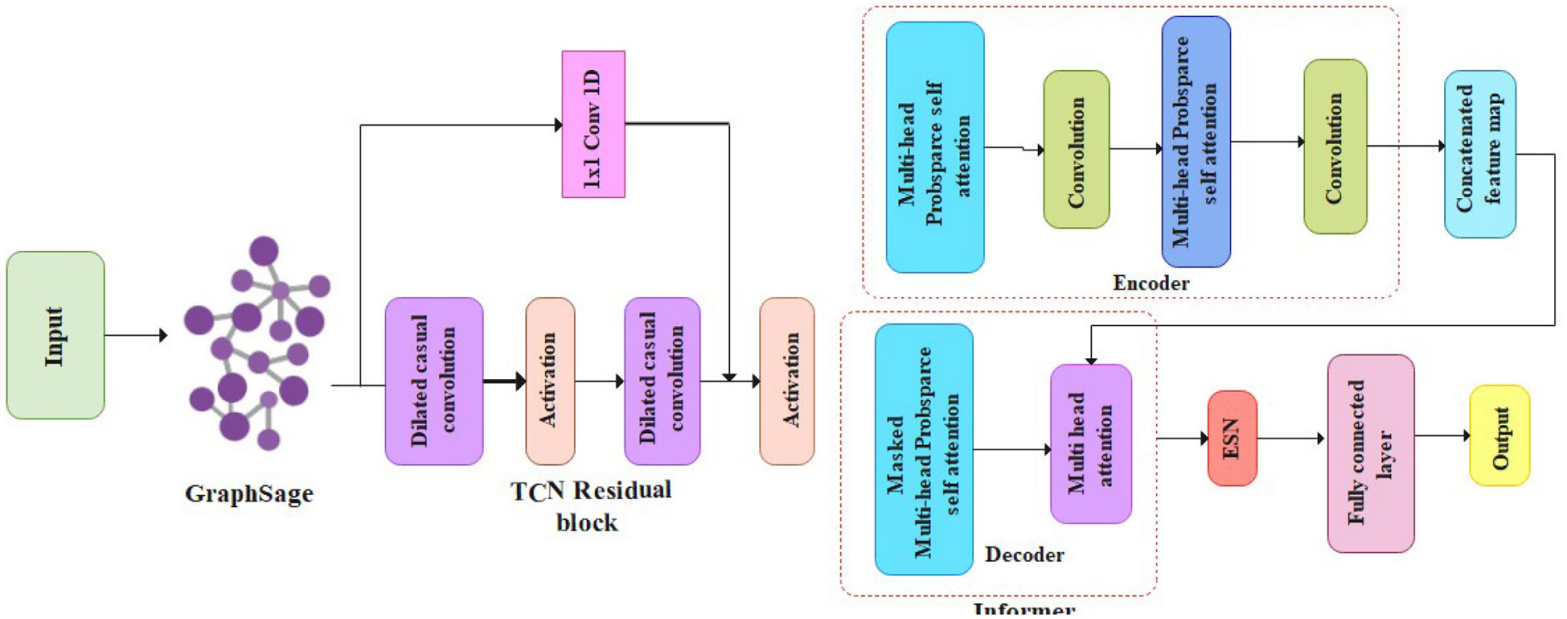
Proposed TSA-HGNN architecture (GraphSAGE → TCN → Informer → ESN → stability regularization → community assignment).

We propose TSA-HGNN, a temporal stability-aware hybrid graph neural network for dynamic community detection in evolving graphs. The framework integrates five complementary components into a unified pipeline: (i) inductive spatial representation learning at each snapshot, (ii) short-range temporal modeling, (iii) efficient long-range temporal dependency learning, (iv) non-linear temporal memory, and (v) stability-aware optimization to regulate unreasonable community drift between adjacent snapshots. In this way, TSA-HGNN is designed to address three key challenges in dynamic community detection: inductive scalability at the snapshot level, effective modeling of both short- and long-horizon temporal dependencies, and stable community evolution over time. Figure 1 illustrates the overall processing pipeline, while the default model configuration is summarized in Table 1.

**Table 1:** Hyperparameter configuration used for TSA-HGNN.

Hyperparameter	Value	Description
GraphSAGE neighbors (*s*)	10	Sampled neighbors per node per layer
GraphSAGE layers	2	Number of aggregation layers
GraphSAGE hidden dim	128	Embedding dimension per node
TCN kernel size (*k*)	3	Dilated causal convolution kernel
TCN dilation schedule	4 layers	$r_{\ell }=2^{\ell }$, ℓ ∈ {0, 1, 2, 3}, giving *r* ∈ {1, 2, 4, 8}
Informer attention heads	8	Multi-head ProbSparse attention
Informer *d*_model_	128	Query/Key/Value dimension
Informer encoder layers	3	Number of stacked encoder blocks
ESN reservoir size (*N*)	500	Number of reservoir neurons
ESN leaky rate (γ)	0.3	Controls state update speed
ESN spectral radius	0.9	Controls stability of reservoir dynamics
Stability weight (λ)	0.1	Balance between stability and accuracy loss
Learning rate	0.001	Adam optimizer
Batch size	32	Graph snapshots per batch
Training epochs	200	With early stopping (patience = 20)
Train / Val / Test split	70 / 10 / 20%	Temporal split to prevent future leakage
Number of runs	5	Independent runs (seeds 1–5)
Total model parameters	~2.1M	Total across all modules

Five independent runs balance computational cost and statistical reliability. The corresponding software versions, hardware specifications, and deterministic settings are provided in the reproducibility checklist in the experimental section.

### 3.1 Dynamic graph snapshot modeling

We represent a dynamic network as an ordered sequence of graph snapshots,


$$\left\{G_{1},G_{2},\ldots ,G_{T}\right\},$$

where each snapshot at time *t* is defined as


$$G_{t}=\left(V_{t},E_{t},{\text{X}}_{t}\right).$$

Here, $V_{t}$ denotes the set of nodes present at snapshot *t*, with $|V_{t}|=n_{t}$. The edge set $E_{t}\subseteq V_{t}\times V_{t}$ encodes the pairwise interactions observed at time *t*, and the corresponding adjacency matrix is ${\text{A}}_{t}\in {\left\{0,1\right\}}^{n_{t}\times n_{t}}$. The node feature matrix ${\text{X}}_{t}\in {\mathbb{R}}^{n_{t}\times d_{\text{in}}}$ assigns a *d*_in_-dimensional attribute vector to each node. In the datasets considered in this study, the node set remains fixed across snapshots (i.e., $V_{t}=V$ for all *t*), while the edge set $E_{t}$ and node features **X**_*t*_ evolve. The framework itself does not assume a fixed node set; this generality is discussed further in Section 3.2.1.

Unlike static community detection, which focuses on partitioning a single graph, dynamic community detection must infer the community structure at each time step while preserving temporal consistency across snapshots. A practical model must therefore capture not only snapshot-level structure, but also how communities evolve through events such as birth, disappearance, merging, splitting, and gradual drift. TSA-HGNN processes snapshots sequentially and employs multi-stage temporal modeling to preserve these dependencies while maintaining stable community assignments over time. A summary of representative methods relevant to dynamic community detection is provided in Table 2.

**Table 2:** Representative methods relevant to dynamic community detection, their core ideas, and limitations in evolving-network settings.

Author(s)/year	Method	Core idea (one-line)	Limitation in dynamic settings
Hamilton et al. (2017)	GraphSAGE	Learns inductive node embeddings via neighborhood sampling and aggregation.	Designed for static graphs; does not model temporal evolution.
Rossi et al. (2020)	TGN	Uses continuous-time message passing with a memory module to encode temporal interactions.	Can incur high memory overhead; limited control over long-range temporal behavior.
Zhao et al. (2020)	T-GCN	Couples spatial encoding with recurrent temporal updates for evolving signals.	RNN-style temporal modeling can be weak for long horizons.
Sankar et al. (2020)	DySAT	Applies self-attention across structural and temporal dimensions to obtain dynamic embeddings.	Often computationally expensive; lacks an explicit stability or smoothness mechanism.
Pareja et al. (2020)	EvolveGCN	Updates GCN parameters over time to reflect network evolution.	Long-horizon performance may become unstable as dynamics accumulate.
Bai et al. (2018)	TCN	Captures temporal patterns using dilated, causal convolutions for sequence learning.	Not inherently graph-structured; requires a graph encoder for topology.
Zhou et al. (2021)	Informer	Reduces Transformer attention cost using ProbSparse attention for long sequences.	Needs adaptation to handle graph-structured inputs and snapshot embeddings.
Jaeger (2001)	ESN	Uses a fixed reservoir with a trained readout to provide nonlinear temporal memory.	Sensitive to reservoir hyperparameters and initialization choices.
Yadati et al. (2019)	HyperGCN	Extends graph convolution to hypergraph structures through a Laplacian-style approximation.	Primarily designed for hypergraph inputs rather than evolving pairwise snapshots.
Berahmand et al. (2025)	OA2H-SP	Uses anchor-adaptive hypergraph learning for efficient community discovery.	Static higher-order formulation; no explicit temporal consistency across snapshots.

#### 3.1.1 Notation and key symbols

To ensure clarity and avoid ambiguity, Table 3 summarizes the key symbols used throughout the methodology. Note in particular that *d* denotes the embedding dimension, while *r* denotes the TCN dilation rate; these are distinct hyperparameters with different meanings.

**Table 3:** Key notation used in the TSA-HGNN methodology.

Symbol	Meaning
*T*	Number of temporal snapshots
*n* or *n*_*t*_	Number of nodes (per snapshot)
*d*	Embedding dimension (GraphSAGE, Informer)
*d*_in_	Input feature dimension
*d*_model_	Informer model dimension (set equal to *d*)
*d*_*y*_	ESN output dimension
*r*	TCN dilation rate (distinct from *d*)
*k*	TCN kernel size
*s*	GraphSAGE neighborhood sample size
*L*	Number of layers (GraphSAGE or TCN depth)
*N*	ESN reservoir size
**S**	Tensor formed from TCN output (Equation 8a)
**Q**, **K**, **V**	Query, key, value matrices (Equations 8b–8d)
**S** ^(*j*)^	Iteratively refined encoder state (Equation 13)
λ	Temporal smoothness weight

### 3.2 Spatial feature extraction using GraphSAGE

Snapshot-level spatial representations are first learned using GraphSAGE, an inductive graph neural model (Hamilton et al., 2017). At each snapshot *t*, GraphSAGE receives the node feature matrix **X**_*t*_ as initial input, setting ${\text{h}}_{u}^{\left(0\right)}={\text{X}}_{t,u}$ for every node $u\in V_{t}$, and uses the adjacency structure defined by $E_{t}$ (equivalently **A**_*t*_) to determine the neighborhood N(u) of each node. The output after *L* layers is a snapshot-level embedding matrix ${\text{Z}}_{t}\in {\mathbb{R}}^{n_{t}\times d}$, where *d* denotes the embedding dimension (a hyperparameter distinct from other model-specific dimensions such as the TCN dilation rate *r* introduced in Section 3.3). Because GraphSAGE operates on (**X**_*t*_, **A**_*t*_) independently at each snapshot, every **Z**_*t*_ depends on the graph structure $G_{t}$ observed at that time step. GraphSAGE avoids full-neighborhood aggregation, which can be expensive on large graphs and less suitable when node sets evolve. Instead, it samples a fixed-size neighborhood for each node, keeping the computation bounded and supporting inductive generalization.

The neighborhood sampling step is


$$\begin{array}{l} N_{s}\left(u\right)=\text{SAMPLE}\left(N\left(u\right),s\right), \end{array}$$ (1)

where N(u) is the full neighborhood of node *u*, *s* is the sampling size, and $N_{s}\left(u\right)$ is the sampled neighborhood.

Neighbor information is aggregated as


$$\begin{array}{l} {\text{h}}_{N_{s}\left(u\right)}^{\left(l\right)}=\text{AGG}(\left\{{\text{h}}_{v}^{\left(l-1\right)}|v\in N_{s}\left(u\right)\right\}), \end{array}$$ (2)

where AGG(·) may denote mean, pooling, or LSTM-based aggregation.

The node embedding update is then given by


$$\begin{array}{l} {\text{h}}_{u}^{\left(l\right)}=\sigma \left([{\text{h}}_{u}^{\left(l-1\right)}∥{\text{h}}_{N_{s}\left(u\right)}^{\left(l\right)}]{\text{W}}^{\left(l\right)}\right), \end{array}$$ (3)

where **W**^(*l*)^ is the trainable weight matrix at layer *l*, σ(·) is a non-linear activation, and ∥ denotes concatenation. Unless otherwise stated, the default sampling size is *s* = 10, as listed in Table 1.

After *L* layers, the final node embedding of node *u* at snapshot *t* is ${\text{Z}}_{t,u}={\text{h}}_{u}^{\left(L\right)}\in {\mathbb{R}}^{d}$, and the full snapshot-level embedding matrix is


$$\begin{array}{l} {\text{Z}}_{t}=[{\text{z}}_{t,1};{\text{z}}_{t,2};\ldots ;{\text{z}}_{t,n_{t}}]\in {\mathbb{R}}^{n_{t}\times d}. \end{array}$$ (3a)

The ordered collection {**Z**_1_, **Z**_2_, …, **Z**_*T*_} forms the temporal embedding sequence that is passed to the downstream temporal modules (TCN in Section 3.3 and Informer in Section 3.4). In the present study, *n*_*t*_ = *n* for all *t*, so the sequence can be compactly represented as a three-dimensional tensor **Z** ∈ ℝ^*T*×*n*×*d*^.

#### 3.2.1 Handling dynamic node sets and directed inputs

A practical dynamic community detection model must accommodate evolving node sets across snapshots. TSA-HGNN handles this naturally through the inductive design of GraphSAGE. When a new node appears at snapshot *t*, its embedding can be generated immediately from its observed features and sampled neighborhood without retraining a transductive embedding table. When a node disappears from the graph, it is simply excluded from the current snapshot and the downstream temporal pipeline. Community merge and split events are not modeled by hand-crafted rules; instead, they are captured implicitly by the temporal modules, which learn how local and global structural changes propagate over time.

When node sets differ between consecutive snapshots, the temporal modules (TCN and Informer) operate on the embedding sequence of each node present in both snapshots. Specifically, for any node $v\in V_{t}\cap V_{t-1}$, the temporal convolutions and attention operations are applied along the temporal axis of that node's embedding trajectory {**z**_1, *v*_, …, **z**_*T, v*_}. Nodes that appear only at a subset of snapshots have shorter trajectories and are padded or excluded as appropriate. In the experimental datasets used in this study, the node set is fixed across all snapshots; thus, this case does not arise in practice. However, the framework does not assume a fixed node set by construction.

**Directed dataset handling (Dataset 2: Reddit Hyperlink Network)**. Since the GraphSAGE aggregation in the present formulation is defined over an undirected neighborhood, the directed adjacency is symmetrized before sampling. Each directed edge (*u*→*v*) is treated as an undirected link (*u, v*), while edge sentiment and other available properties are preserved through aggregated node-level attributes. Equations 1–3 define the GraphSAGE neighborhood sampling, aggregation, and node embedding update steps. This simplification enables a consistent inductive encoder across datasets, though it discards edge-direction semantics. A direction-aware encoder with separate incoming and outgoing aggregation is a natural extension for future work.

### 3.3 Short-range temporal modeling using TCN residual blocks

To capture local temporal evolution across consecutive snapshots, TSA-HGNN applies a TCN over the sequence of snapshot embeddings (Bai et al., 2018). TCNs support parallel computation, stable gradient flow, and causal modeling without future leakage. By combining causal and dilated convolutions, the temporal receptive field expands efficiently across multiple snapshots.

For a kernel of size *k* and dilation rate *r*, the required zero-padding is


$$\begin{array}{l} p=\left(k-1\right)r, \end{array}$$ (4)

with the dilation rate typically increasing as $r_{\ell }=2^{\ell }$ at layer ℓ, for ℓ ∈ {0, 1, 2, 3}.

Let **Z** = {**Z**_1_, **Z**_2_, …, **Z**_*T*_} denote the temporal sequence of snapshot embeddings produced by GraphSAGE (Section 3.2), where *T* is the number of snapshots as defined in Section 3.1. A dilated causal convolution at layer *L* is


$$\begin{array}{l} {\text{F}}_{r}^{L}\left(t\right)=\overset{k-1}{\underset{i=0}{\sum }}f_{i}{\text{Z}}_{t-ir}^{\left(L-1\right)}, \end{array}$$ (5)

where *f*_*i*_ denotes the *i*-th learned convolutional filter coefficient of the kernel, and ${\text{Z}}_{t-ir}^{\left(L-1\right)}$ is the embedding at temporal position *t*−*ir* in the previous layer. The activation is


$$\begin{array}{l} {\text{H}}_{r}^{L}\left(t\right)=\text{ReLU}({\text{F}}_{r}^{L}\left(t\right)+b), \end{array}$$ (6)

where *b* is a bias term and


$$\text{ReLU}(t)=\max(0,t)=\{\begin{array}{ll} 0, & t\leq 0,\\ t, & t>0. \end{array}$$ (7)

Equations 4–7 define the TCN padding, dilated causal convolution, activation, and ReLU operations.

Residual connections are used to stabilize deeper temporal modeling:


$$\begin{array}{l} {\tilde{\text{H}}}_{r}^{L}\left(t\right)=\text{ReLU}({\text{F}}_{r}^{L}\left(t\right)+b+{\text{H}}_{r}^{\left(L-1\right)}\left(t\right)). \end{array}$$ (8)

This component captures short-range temporal changes, such as small edge perturbations, local reorganization, and immediate community transitions between adjacent snapshots.

#### 3.3.1 Multi-scale temporal design rationale

Short- and long-range temporal dependencies are not equally expressed in evolving graphs. Some changes arise from local structural updates over one or two snapshots, whereas others emerge gradually over longer horizons. Therefore, TSA-HGNN adopts a multi-scale design rather than relying on a single temporal module. The TCN captures local and short-horizon evolution efficiently, while the Informer models longer-range temporal dependencies at lower attention cost than dense temporal self-attention. This combination allows the framework to capture complementary temporal patterns while preserving computational efficiency.

### 3.4 Long range dependency learning using informer

While the TCN captures local temporal behavior, dynamic community evolution may also depend on patterns that unfold over much longer time spans. To model these long-horizon dependencies efficiently, TSA-HGNN employs an Informer-based encoder and decoder (Zhou et al., 2021). The Informer receives as input the TCN output sequence $\left\{{\tilde{\text{H}}}_{r}^{L}\left(1\right),\ldots ,{\tilde{\text{H}}}_{r}^{L}\left(T\right)\right\}$ produced by the residual blocks described in Section 3.3, reshaped as a tensor **S** ∈ ℝ^*n*×*T*×*d*^ where *n* is the number of nodes, *T* is the number of snapshots, and *d* is the embedding dimension. Within the Informer, each node's temporal trajectory of length *T* is treated as a sequence, and ProbSparse attention operates along the temporal axis. This arrangement allows the TCN to first refine local temporal patterns over short horizons, after which the Informer captures longer-range dependencies that span multiple snapshots. Informer replaces dense attention with ProbSparse attention, thereby reducing the computational burden while retaining long-range modeling ability. Positional encoding is used to preserve temporal order, and masked attention is used to prevent future leakage.

The tensor **S** is constructed by stacking the TCN output embeddings:


$$\begin{array}{l} \text{S}=\text{reshape}(\left\{{\tilde{\text{H}}}_{r}^{L}\left(1\right),\ldots ,{\tilde{\text{H}}}_{r}^{L}\left(T\right)\right\})\in {\mathbb{R}}^{n\times T\times d}, \end{array}$$ (8a)

where each slice ${\text{S}}_{:,t,:}\in {\mathbb{R}}^{n\times d}$ corresponds to the refined embedding at snapshot *t*. The query, key, and value tensors for the Informer attention mechanism are obtained via learned linear transformations:


$$\begin{array}{l} \text{Q}=\text{S}{\text{W}}_{Q}, \end{array}$$ (8b)

$$\begin{array}{l} \text{K}=\text{S}{\text{W}}_{K}, \end{array}$$ (8c)

$$\begin{array}{l} \text{v}=\text{S}{\text{W}}_{V}, \end{array}$$ (8d)

where ${\text{W}}_{Q},{\text{W}}_{K},{\text{W}}_{V}\in {\mathbb{R}}^{d\times d}$ are trainable projection matrices. In this implementation, we set *d*_model_ = *d* for computational efficiency, so the attention mechanism operates directly on the embedding dimension.

Let the query, key, and value vectors be **Q**, **K**, and **V**. For a query **q**_*i*_, the attention output is


$$\begin{array}{l} A\left({\text{q}}_{i},\text{K},\text{v}\right)=\underset{j}{\sum }\left(\frac{\kappa \left({\text{q}}_{i},{\text{K}}_{j}\right)}{\sum_{l}\kappa \left({\text{q}}_{i},{\text{K}}_{l}\right)}\right){\text{v}}_{j}={\text{𝔼}}_{p\left({\text{K}}_{j}|{\text{q}}_{i}\right)}\left[{\text{v}}_{j}\right], \end{array}$$ (9)

where κ(·, ·) is the similarity kernel.

Query sparsity is measured as


$$\begin{array}{l} M\left({\text{q}}_{i},\text{K}\right)=\ln\left(\overset{L_{k}}{\underset{j=1}{\sum }}\exp\left(\frac{{\text{q}}_{i}{\text{K}}_{j}^{⊤}}{\sqrt{d_{\text{model}}}}\right)\right)-\frac{1}{L_{k}}\overset{L_{k}}{\underset{j=1}{\sum }}\frac{{\text{q}}_{i}{\text{K}}_{j}^{⊤}}{\sqrt{d_{\text{model}}}}, \end{array}$$ (10)

and the attention operation is


$$\begin{array}{l} A\left(\text{Q},\text{K},\text{v}\right)=\text{softmax}\left(\frac{\bar{\text{Q}}{\text{K}}^{⊤}}{\sqrt{d_{\text{model}}}}\right)\text{v}, \end{array}$$ (11)

where $\bar{\text{Q}}$ denotes the selected dominant queries under ProbSparse selection.

Layer normalization is written as


$$\begin{array}{l} \text{O}=\text{LayerNorm}(\text{x}+\text{Sublayer}\left(\text{x}\right)), \end{array}$$ (12)

and Informer distillation along the temporal dimension progressively refines the encoder state **S**^(*j*)^ as:


$$\begin{array}{l} {\text{S}}^{\left(j+1\right)}=\text{MaxPool}\left(\text{ELU}\left(\text{Conv1d}\left({\left[{\text{S}}^{\left(j\right)}\right]}_{AB}\right)\right)\right), \end{array}$$ (13)

where **S**^(0)^ = **S** is the initial encoder input defined in Equation 8a, and ${\left[{\text{S}}^{\left(j\right)}\right]}_{AB}$ denotes the attention-block output at encoder layer *j*.

In TSA-HGNN, Informer is applied to the sequence of snapshot embeddings generated by the previous stages, enabling efficient long-range modeling of community evolution without quadratic attention cost in *T*. The tensor **S** is first defined and constructed from the TCN output (Equation 8a), then used to derive the query, key, and value matrices **Q**, **K**, and **V** (Equations 8b–8d). The Informer encoder then processes these representations through three stacked self-attention and distillation layers (Equations 9–13), where the distillation operation (Equation 13) progressively refines the encoder state **S**^(*j*)^ initialized from **S**. The Informer decoder then combines masked self-attention with cross-attention over the encoder output to produce the long-range temporal representation **R** ∈ ℝ^*n*×*T*×*d*^. Each slice ${\text{R}}_{:,t,:}\in {\mathbb{R}}^{n\times d}$ is the Informer output at snapshot *t*, which is subsequently passed to the ESN module described in Section 3.5. The kernel κ(·, ·) in Equation 9 denotes the exponential similarity kernel $\kappa \left(\text{q},\text{K}\right)=\exp\left(\text{q}{\text{K}}^{⊤}/\sqrt{d_{\text{model}}}\right)$, where *d*_model_ = *d* as stated in Equations 8b–8d. The symbol *L*_*k*_ in Equation 10 denotes the key sequence length (equal to *T*), and $\bar{\text{Q}}$ in Equation 11 denotes the subset of queries selected by the ProbSparse criterion with the highest sparsity scores *M*(**q**_*i*_, **K**).

### 3.5 Non-linear reservoir memory using echo state network

**Figure 2:**
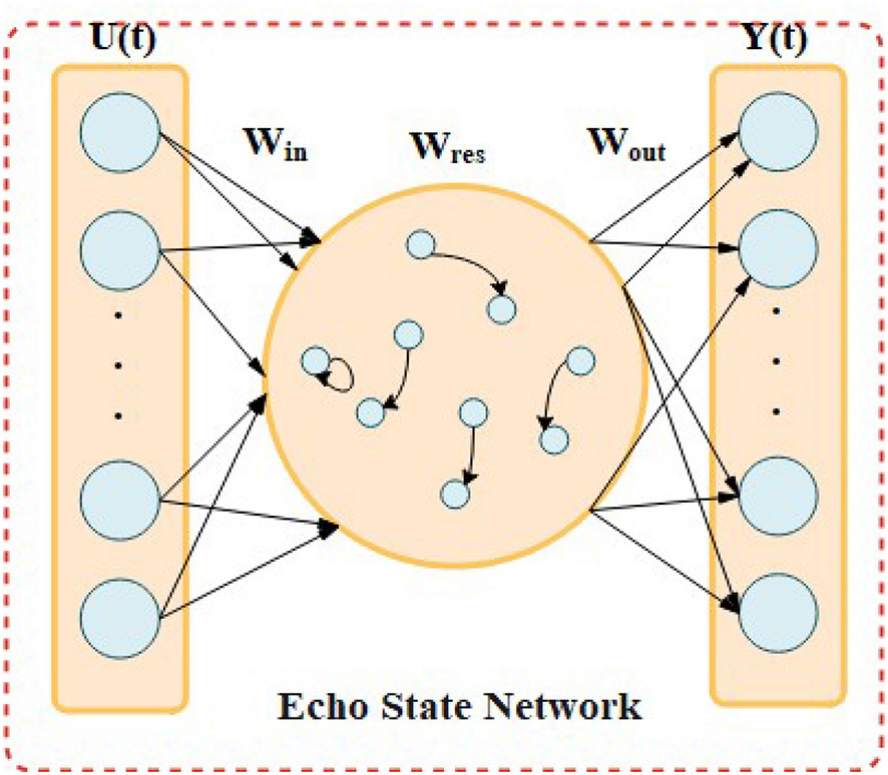
Echo State Network (ESN) reservoir and readout.

To provide additional non-linear temporal memory with low training overhead, an ESN is placed after the Informer decoder (Jaeger, 2001). In an ESN, the reservoir connections are fixed after initialization, and only the readout layer is trained. Figure 2 illustrates the internal structure of the ESN.

Let ${\text{R}}_{:,t,:}\in {\mathbb{R}}^{n\times d}$ denote the Informer output at snapshot *t*. The vectorized representation ${\text{r}}_{t}=\text{vec}\left({\text{R}}_{:,t,:}\right)\in {\mathbb{R}}^{nd\times 1}$ serves as input to the ESN fusion block. Define ${\text{W}}_{in}\in {\mathbb{R}}^{N\times nd}$ as the input-to-reservoir weights, ${\text{W}}_{res}\in {\mathbb{R}}^{N\times N}$ as the recurrent reservoir weights, and ${\text{W}}_{out}\in {\mathbb{R}}^{d_{y}\times N}$ as the readout weights, where *d*_*y*_ is the ESN output dimension. The ESN receives **r**_*t*_ as input **u**(*t*) = **r**_*t*_. A leaky ESN state update is given by


$$\begin{array}{l} \text{x}\left(t\right)=\left(1-\gamma \right)\text{x}\left(t-1\right)+\gamma \tanh({\text{W}}_{in}\text{u}\left(t\right)+{\text{W}}_{res}\text{x}\left(t-1\right)), \end{array}$$ (14)

where **x**(*t*) ∈ ℝ^*N*×1^ is the reservoir state and γ ∈ (0, 1) is the leaky rate.

The ESN readout is:


$$\begin{array}{l} \text{y}\left(t\right)={\text{W}}_{out}\text{x}\left(t\right)\in {\mathbb{R}}^{d_{y}\times 1}. \end{array}$$ (14a)

Equations 14, 15 define the ESN state update and fused temporal representation. The ESN output is concatenated with the vectorized Informer representation:


$$\begin{array}{l} {\text{h}}_{t}=[\text{y}\left(t\right)∥{\text{r}}_{t}]\in {\mathbb{R}}^{\left(d_{y}+nd\right)\times 1}. \end{array}$$ (15)

The final stabilized embedding ${\hat{\text{Z}}}_{t}$ is obtained via a learned projection and reshaping:


$$\begin{array}{l} {\hat{\text{Z}}}_{t}={\text{W}}_{p}{\text{h}}_{t}+{\text{b}}_{p}\in {\mathbb{R}}^{nd\times 1}, \end{array}$$ (15a)

$$\begin{array}{l} {\hat{\text{Z}}}_{t}=\text{reshape}\left({\hat{\text{Z}}}_{t}\right)\in {\mathbb{R}}^{n\times d}, \end{array}$$ (15b)

where ${\text{W}}_{p}\in {\mathbb{R}}^{nd\times \left(d_{y}+nd\right)}$ and ${\text{b}}_{p}\in {\mathbb{R}}^{nd\times 1}$ are trainable parameters. This embedding is used for temporal prediction (Equation 18) and community assignment (Section 3.7).

This fused temporal embedding is then projected to produce the final community-oriented representation.

#### 3.5.1 ESN vs. trainable recurrent memory

The motivation for using ESN is not only its temporal expressiveness, but also computational efficiency. Unlike GRU and LSTM, ESN does not require full backpropagation through time over the recurrent state transitions because only the readout is trained. This reduces the number of trainable parameters and lowers the temporal optimization burden. In the present framework, ESN serves as a lightweight non-linear memory module that complements the TCN and Informer rather than replacing them. An empirical comparison with GRU- and LSTM-based alternatives is provided later in the experimental section.

### 3.6 Stability regularization and training objective

Dynamic community detection should not produce abrupt changes in assignments unless the observed network evolution strongly supports them. To discourage unreasonable snapshot-to-snapshot variation, a temporal smoothness constraint is introduced:


$$\begin{array}{l} L_{stability}=\overset{T}{\underset{t=2}{\sum }}||{\text{Z}}_{t}-{\text{Z}}_{t-1}{||}_{F}^{2}, \end{array}$$ (16)

where ${\text{Z}}_{t}\in {\mathbb{R}}^{|V_{t}|\times d}$ denotes the node embedding matrix at snapshot *t*, and ||·||_*F*_ is the Frobenius norm.

A reconstruction loss is computed using binary cross-entropy between the observed adjacency **A**_*t*_ and reconstructed probabilities ${\hat{\text{A}}}_{t}$. The reconstruction is performed using the GraphSAGE embedding **Z**_*t*_, which captures the structural relationships of the graph at snapshot *t* before temporal aggregation.

The reconstructed adjacency is obtained from the embedding matrix **Z**_*t*_ via an inner-product decoder followed by a sigmoid activation:


$$\begin{array}{l} {\hat{\text{A}}}_{t}\left(u,v\right)=\sigma ({\text{Z}}_{t,u}^{⊤}{\text{Z}}_{t,v}), \end{array}$$ (16a)

where σ(·) denotes the sigmoid function, ${\text{Z}}_{t,u}\in {\mathbb{R}}^{d}$ is the embedding of node *u* at snapshot *t*, and ${\hat{\text{A}}}_{t}\in {\left[0,1\right]}^{n_{t}\times n_{t}}$ is the full reconstructed adjacency matrix. This definition allows the reconstruction loss to measure how faithfully the learned embeddings preserve the observed graph structure at each snapshot. The reconstruction loss is then:


$$\begin{array}{l} L_{recon}\left(t\right)=-\frac{1}{|V_{t}{|}^{2}}\underset{u\in V_{t}}{\sum }\underset{v\in V_{t}}{\sum }({\text{A}}_{t}\left(u,v\right)\log{\hat{\text{A}}}_{t}\left(u,v\right)\\ \;+\left(1-{\text{A}}_{t}\left(u,v\right)\right)\log\left(1-{\hat{\text{A}}}_{t}\left(u,v\right)\right)). \end{array}$$ (17)

Equation 17 defines the reconstruction loss used to preserve the observed graph structure.

A temporal modeling loss penalizes the discrepancy between the predicted embedding of the next snapshot and the actual embedding:


$$\begin{array}{l} L_{temporal}=\frac{1}{T-1}\overset{T-1}{\underset{t=1}{\sum }}||{\hat{\text{Z}}}_{t+1}-{\text{Z}}_{t+1}{||}_{F}^{2}. \end{array}$$ (18)

Equation 19 defines the overall training objective is


$$\begin{array}{l} L_{total}=L_{recon}+L_{temporal}+\text{λ}L_{stability}, \end{array}$$ (19)

where λ controls the strength of the stability constraint.

#### 3.6.1 Theoretical justification for the smoothness penalty

The smoothness term in Equation (16) is introduced to reduce unnecessary changes in the learned embedding space across adjacent snapshots. Its purpose is to discourage embedding trajectories that would induce unstable community assignments in the absence of corresponding structural evidence from the evolving graph sequence.

##### 3.6.1.1 Theorem 1 (stability–switch rate bound)

Let the community assignment at snapshot *t* be defined by


$$\begin{array}{l} c_{t}\left(v\right)=\arg\underset{k}{\min}||{\text{Z}}_{t,v}-\mu_{k}{||}_{2}, \end{array}$$ (20)

where **μ**_*k*_ denotes the centroid of community *k*. Assume that the decision boundaries induced by the clustering rule are Lipschitz continuous with constant *L*, and let δ>0 denote the minimum inter-cluster margin. Then, the expected community switch rate satisfies the upper bound


$$\begin{array}{l} E\left[{\text{SwitchRate}}_{t}\right]\leq \frac{L}{\delta^{2}}L_{stability}, \end{array}$$ (21)

where


$$\begin{array}{l} L_{stability}=||{\text{Z}}_{t}-{\text{Z}}_{t-1}{||}_{F}^{2}. \end{array}$$ (22)

##### 3.6.1.2 Proof sketch

A node *v* changes its assigned community between snapshots *t*−1 and *t* only if its embedding crosses a decision boundary separating two clusters. Under the Lipschitz continuity assumption, such a crossing requires the embedding displacement to be sufficiently large, namely


$$\begin{array}{l} ||{\text{Z}}_{t,v}-{\text{Z}}_{t-1,v}{||}_{2}\geq \frac{\delta }{L}. \end{array}$$ (23)

Summing this condition over all nodes shared by consecutive snapshots and applying Markov's inequality yields an upper-bound style relationship between the fraction of switched nodes and the total embedding displacement measured by the Frobenius norm. Since $L_{stability}$ aggregates these squared embedding displacements, minimizing it directly reduces an upper bound on the expected switch rate. Equations 20–23 define the community assignment rule, the switch-rate bound, the stability term, and the margin condition used in the theoretical justification.

##### 3.6.1.3 Interpretation

The result provides theoretical intuition for why the smoothness penalty promotes stable community evolution: smaller snapshot-to-snapshot embedding movement implies a lower probability of discrete community reassignment. This theorem should be interpreted as an upper-bound style justification for the stability regularizer, rather than as a complete convergence guarantee for the full optimization pipeline. The empirical switch-rate analysis in Section 4 is included to verify this connection quantitatively.

#### 3.7 Community assignment

Given the stabilized embedding ${\hat{\text{Z}}}_{t}\in {\mathbb{R}}^{|V_{t}|\times d}$, community labels are obtained using *k*-means clustering:


$$\begin{array}{l} c_{t}\left(u\right)=\arg\underset{k}{\min}||{\hat{\text{Z}}}_{t,u}-\mu_{k}{||}_{2}^{2}, \end{array}$$ (24)

Equation 24 defines the final community assignment rule. Where ${\hat{\text{Z}}}_{t,u}$ is the embedding of node *u* at snapshot *t* and **μ**_*k*_ is the centroid of community *k*. The number of communities *K* is treated as a dataset-specific hyperparameter. Because stability is enforced in the continuous embedding space before clustering, smoother embeddings tend to yield more consistent discrete assignments across consecutive snapshots.

#### 3.8 Computational complexity

Let |V| denote the number of nodes per snapshot, *T* the number of snapshots, *s* the GraphSAGE neighborhood sample size, *d* the embedding dimension, *k* the TCN kernel size, and *N* the ESN reservoir size. GraphSAGE has per-snapshot complexity O(|V|sd) under fixed neighborhood sampling. TCN across *T* snapshots contributes O(T|V|kd). Informer with ProbSparse attention scales as O(T|V|logT) with respect to the temporal horizon *T*, rather than the $O\left(T^{2}|V|\right)$ cost of dense attention. ESN updates contribute O(TNd), and stability regularization adds O(T|V|d). Therefore, the overall complexity is


$$\begin{array}{l} O\left(T|V|\left(s+k\right)d+T|V|\log T+TNd\right), \end{array}$$ (25)

which remains sub-quadratic in *T* under ProbSparse attention.

#### 3.9 Baseline selection and fair-comparison notes

Baselines were selected to represent complementary method families with stable, publicly available implementations that can be applied consistently under the snapshot-based evaluation protocol. Equation 25 summarizes the overall computational complexity of TSA-HGNN. DySAT (Sankar et al., 2020) relies on temporal self-attention under a fixed-node assumption, which is not directly compatible with the evolving node sets considered in this work without modifying the method or the protocol. Methods introduced very recently, such as STEC-Net (Xu and Chu, 2025), GANSE (Liu X. et al., 2024), and CL-OND (Li et al., 2025), do not yet offer stable public implementations with clear configuration parity under the same snapshot-based regime. Therefore, they are discussed in the literature review and considered relevant future points of comparison, but they are not included as primary reproducible baselines in the present experimental study. The baseline inclusion and adaptation protocol used in this study is summarized in Table 4.

**Table 4:** Baseline inclusion and fair-comparison protocol used in this study.

Method family	Native setting	Main compatibility issue	Use in this study
DeepWalk/Node2Vec	Static pairwise graphs	No temporal modeling	Included as static embedding baselines
TGN/TGAT/GCN-LSTM	Dynamic or temporal graph learning	Require adaptation to a common snapshot protocol for fair comparison	Included as primary temporal baselines
DySAT	Dynamic graphs with fixed node-set assumption	Not directly compatible with evolving node sets without protocol modification	Discussed, but excluded from direct primary comparison
STEC-Net/GANSE / CL-OND	Recent dynamic community detection methods	No stable public implementation with configuration parity under the same snapshot protocol	Discussed in the literature review; treated as future reproducible comparisons
Higher-order/hypergraph methods	Static or alternative higher-order graph settings	Not natively formulated for evolving pairwise snapshots	Included only through explicit adapted comparison protocol

#### 3.10 Higher-order baseline adaptation and fair comparison

More recent higher-order and hypergraph-based methods provide a useful complementary perspective, as they model multi-way relations more explicitly than standard pairwise graph methods. However, these methods are not natively formulated for the same evolving pairwise snapshot setting used in TSA-HGNN. Therefore, any comparison with higher-order baselines must be interpreted carefully. In the present study, higher-order baselines are included only when they can be adapted to the snapshot-based evaluation protocol in a transparent and reproducible way. This adaptation is intended to provide a fair reference point rather than to claim exact equivalence of problem formulation. The main comparison logic used for baseline inclusion, exclusion, and adaptation is summarized in Table 4.

## 4 Results and discussion

This section evaluates the performance of TSA-HGNN for dynamic community detection in evolving graphs. The experimental study is designed to assess four aspects of the proposed framework: (i) structural community quality, (ii) clustering validity, (iii) temporal stability across adjacent snapshots, and (iv) computational efficiency. We compare TSA-HGNN with representative static embedding methods and temporal graph learning baselines under a common snapshot-based protocol, with TGAT (Xu et al., 2020) treated as the strongest baseline for primary statistical comparison. Unless otherwise stated, all reported values are mean ± standard deviation over five independent runs with random seeds {1, 2, 3, 4, 5}.

### 4.1 Dataset description

The benchmark suite covers controlled synthetic dynamics, temporal interaction networks, and large-scale collaboration graphs. A summary of the datasets used in this study is provided in Table 5.

**Table 5:** Summary of datasets used in the experimental evaluation.

Dataset	Nodes	Edges	Snapshots	Graph type	Label source
Dataset 1 (LFR/micro-networks)	Varies	Varies	10/20	Undirected	Ground truth
Dataset 2 (Reddit Hyperlink)	Large-scale	Temporal	10	Directed	Proxy metadata labels
Dataset 3 (DBLP Collaboration)	317,080	1,049,866	10	Undirected	Proxy venue labels
CollegeMsg-style surrogate	200	Synthetic temporal	10	Temporal surrogate	Planted communities

**Dataset 1: Complex Network Community Detection Dataset**. This benchmark consists of LFR synthetic graphs and two classical micro-networks with known community structures, namely Zachary's Karate Club and Dolphins. LFR graphs are widely used for evaluating community detection because they allow controlled variation in the degree distribution, community size, and the mixing parameter μ, while preserving the reference labels. Each LFR graph is treated as a single snapshot and ordered by increasing graph complexity, yielding *T* = 20 time-ordered snapshots. For Karate and Dolphins, we generate *T* = 10 perturbed snapshots by rewiring 5% of the edges at each step following the protocol of Sattar et al. (2023). This dataset, therefore, provides a controlled setting in which both community quality and temporal stability can be evaluated against ground-truth communities.

**Dataset 2: Reddit Hyperlink Network**. This dataset is a large directed temporal interaction graph of subreddits, where nodes correspond to subreddits, and directed edges represent hyperlinks between them over time. The time-stamped edges also carry sentiment and text-derived attribute information. For snapshot-based evaluation, the interaction stream is segmented into time-ordered graph snapshots $G_{t}$. Since verified communities are not directly available, subreddit metadata is used as reference or proxy labels for reporting supervised-style metrics such as Accuracy, F-Score, NMI, and ARI. Conversely, Modularity *Q* is computed solely from the graph structure and is treated as the most faithful structure-based indicator on this dataset.

**Dataset 3: DBLP Collaboration Network**. DBLP is a large co-authorship graph with 317,080 nodes and 1,049,866 edges. We construct temporal snapshots using non-overlapping three-year publication windows from 1990 to 2020, resulting in *T* = 10 snapshots (Yuan et al., 2025). An edge is included in snapshot $G_{t}$ if the earliest publication associated with that collaboration falls in the corresponding time window. Venue-based metadata is used to build reference or proxy labels for snapshot-level evaluation.

**Additional temporal benchmark**. To broaden the experimental scope, we also include a CollegeMsg-style temporal surrogate with planted community structure and timestamped interactions. This benchmark is designed to mimic realistic messaging network dynamics while retaining a known community structure for a controlled comparison. It serves as an additional test of temporal robustness beyond the original three datasets.

**Ground-truth and reference labels**. Dataset 1 uses ground-truth community labels. For the remaining datasets, reference or proxy labels are used only for evaluation and not for training, model selection, or hyperparameter tuning. For Reddit, Modularity *Q* remains the main structure-preserving metric because metadata-derived labels only approximate latent community structure. For DBLP, venue-based labels serve as reference labels for reporting supervised-style clustering metrics.

### 4.2 Implementation details

All experiments were conducted on a workstation with an NVIDIA RTX 3090 GPU (24 GB VRAM), an Intel Core i9-10900X CPU, and 64 GB RAM. The implementation uses Python 3.9, PyTorch 2.0, and PyTorch Geometric 2.3. We train all models for up to 200 epochs with early stopping based on validation performance. In practice, the models converge after approximately 87 ± 12, 94 ± 9, and 112 ± 15 epochs for Datasets 1, 2, and 3, respectively. The default hyperparameter setting is summarized in Table 1.

For all datasets, we adopt a chronological split with 70% of the snapshots used for training, 10% for validation, and 20% for testing. This ensures that no future information is used to predict earlier graph states. For Dataset 1 with *T* = 20, the split yields 14 training snapshots, 2 validation snapshots, and 4 test snapshots. For Datasets 2 and 3 with *T* = 10, the split yields 7 training snapshots, 1 validation snapshot, and 2 test snapshots. Because the number of held-out test snapshots is limited for some datasets, especially Datasets 2 and 3, metric variance and statistical power must be interpreted with appropriate caution.

The stability weight λ is selected from {0.05, 0.10, 0.20} based on validation modularity, and the default value is set to λ = 0.10. The ESN spectral radius is selected from {0.7, 0.9, 0.95}, with 0.9 adopted as the best trade-off between memory retention and stable reservoir dynamics. The reservoir size is selected from {200, 500, 1000} and set to *N* = 500. The remaining implementation settings follow the standard configurations of GraphSAGE (Hamilton et al., 2017), TCN (Bai et al., 2018), and Informer (Zhou et al., 2021).

To quantify temporal stability directly, we measure the *community switch rate*, defined at time *t* as


$$\begin{array}{l} {\text{SwitchRate}}_{t}=\frac{1}{\left|V_{t}\cap V_{t-1}\right|}\underset{v\in V_{t}\cap V_{t-1}}{\sum }\text{1}\left[c_{t}\left(v\right)\neq c_{t-1}\left(v\right)\right], \end{array}$$ (26)

where *c*_*t*_(*v*) denotes the community assigned to node *v* at snapshot *t*. Lower switch-rate values indicate more stable community evolution.

### 4.3 Performance analysis on dataset 1

**Figure 3:**
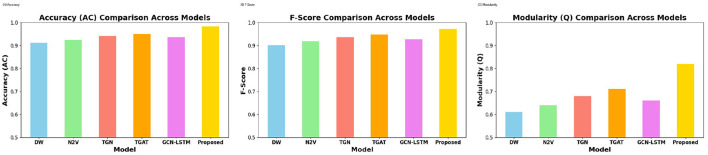
Dataset 1: comparison of Accuracy (AC), F-Score, and Modularity (*Q*) across models.

**Figure 4:**
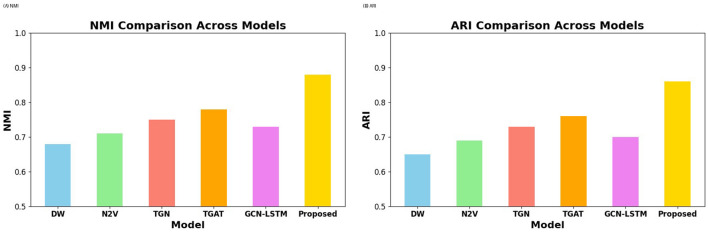
Dataset 1: comparison of NMI and ARI across models.

The performance of TSA-HGNN and the competing methods on Dataset 1 is shown in Figures 3, 4. Figure 3 compares Accuracy, F-Score, and Modularity, while Figure 4 compares NMI and ARI. Additional reproducibility details are provided in Appendix A.

**Overall performance**. TSA-HGNN achieves the strongest results on Dataset 1, with Accuracy = 0.9843, F-Score = 0.9720, Modularity *Q* = 0.8200, NMI = 0.8800, and ARI = 0.8600. These values are consistently higher than those of TGAT (Xu et al., 2020), TGN (Rossi et al., 2020), GCN-LSTM (Seo et al., 2018), Node2Vec (Grover and Leskovec, 2016), and DeepWalk (Perozzi et al., 2014). In particular, the margin in Modularity over TGAT and the static baselines indicates that the proposed combination of spatial encoding, multi-scale temporal modeling, and stability-aware optimization yields more coherent, better-separated communities in evolving graphs.

**Figure 5:**
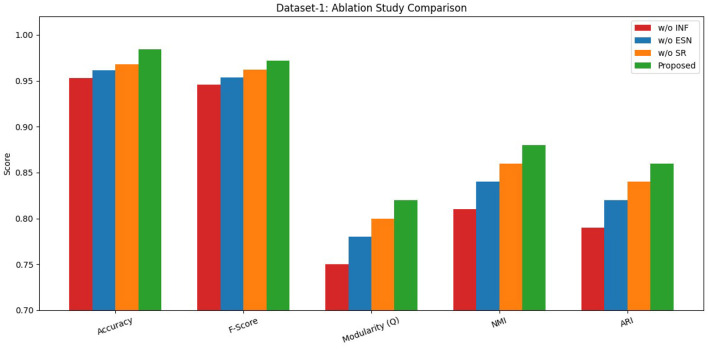
Dataset 1: ablation study comparison across key variants.

**Ablation analysis**. The ablation results for Dataset 1 are summarized in Table 6 and illustrated in Figure 5. The full model performs best across all five metrics. Removing the temporal smoothness regularization reduces both clustering quality and stability. Removing the ESN reservoir also yields weaker results, indicating that lightweight non-linear memory contributes to temporal representation quality. Equation 26 defines the community switch-rate metric used to evaluate temporal stability. The most pronounced degradation occurs when the Informer component is removed, which confirms that long-range temporal dependency modeling is especially important for tracking community evolution over time. Removing TCN weakens sensitivity to local temporal reorganization, whereas replacing GraphSAGE with an MLP yields the largest drop in structural quality, underscoring the importance of graph-aware spatial encoding.

**Table 6:** Dataset 1 ablation results (mean ± std over 5 runs).

Variant	AC	F-Score	*Q*	NMI	ARI
Proposed (Full)	0.9843 ± 0.003	0.9720 ± 0.003	0.8200 ± 0.005	0.8800 ± 0.005	0.8600 ± 0.005
w/o SR	0.9680 ± 0.004	0.9620 ± 0.004	0.8000 ± 0.007	0.8600 ± 0.007	0.8400 ± 0.007
w/o ESN	0.9615 ± 0.004	0.9540 ± 0.004	0.7800 ± 0.007	0.8400 ± 0.007	0.8200 ± 0.007
w/o TCN	0.9580 ± 0.004	0.9490 ± 0.004	0.7700 ± 0.007	0.8300 ± 0.007	0.8080 ± 0.007
w/o INF	0.9530 ± 0.004	0.9460 ± 0.004	0.7500 ± 0.007	0.8100 ± 0.007	0.7900 ± 0.007
w/o GraphSAGE (MLP)	0.9480 ± 0.005	0.9390 ± 0.005	0.7510 ± 0.008	0.8030 ± 0.008	0.7920 ± 0.008

### 4.4 Performance analysis on dataset 2

**Figure 6:**
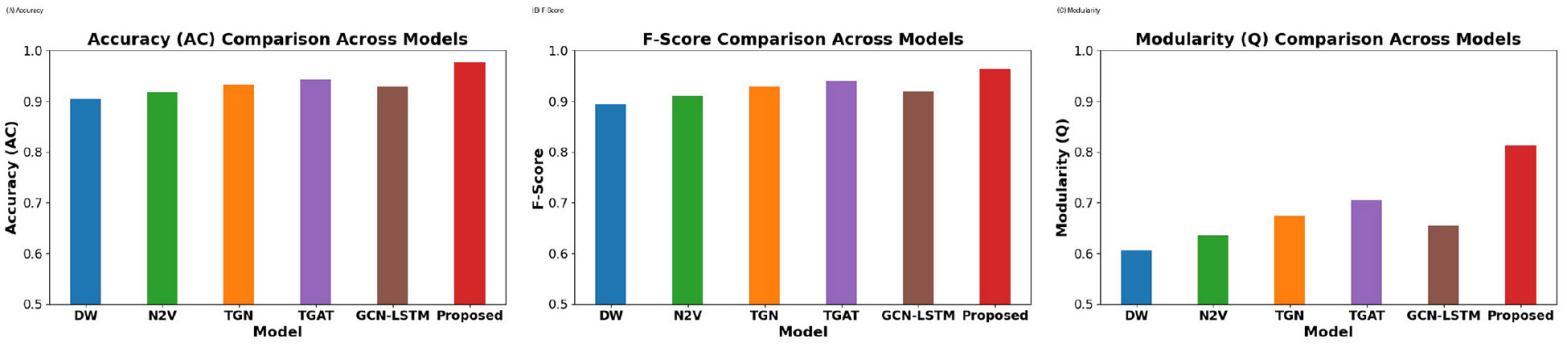
Dataset 2: comparison of Accuracy (AC), F-Score, and Modularity (*Q*) across models.

**Figure 7:**
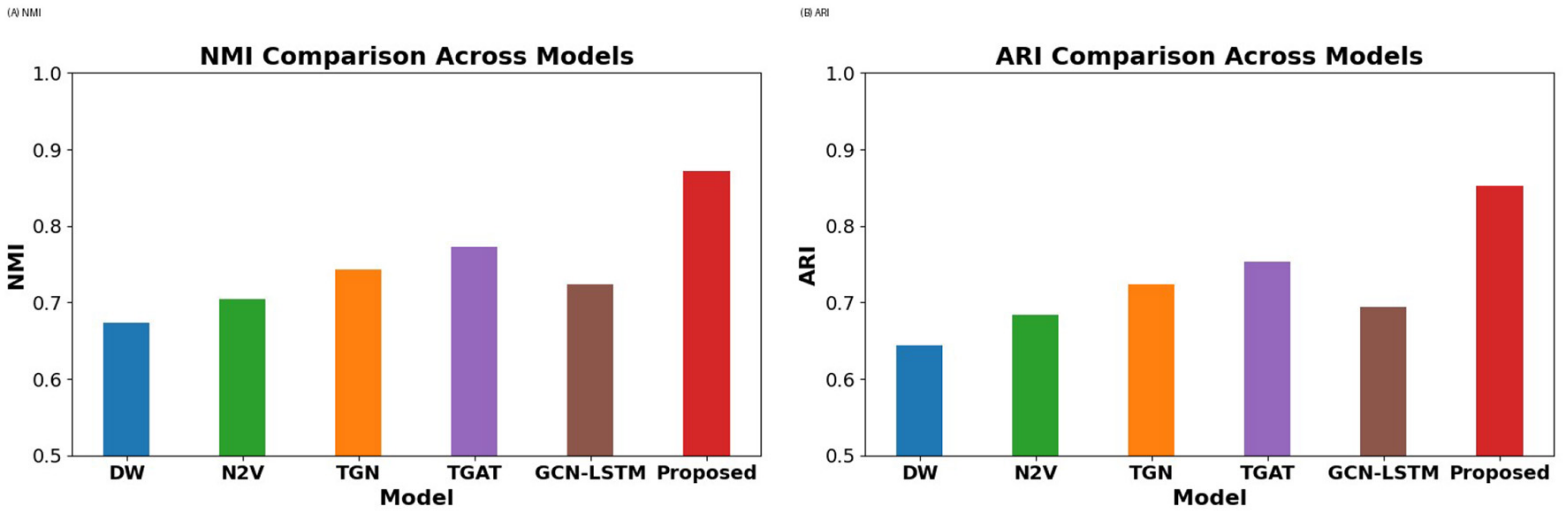
Dataset 2: comparison of NMI and ARI across models.

The comparative results on Dataset 2 are presented in Figures 6, 7. Figure 6 reports Accuracy, F-Score, and Modularity, while Figure 7 reports NMI and ARI.

**Overall performance**. TSA-HGNN again achieves the strongest overall performance across all evaluation metrics. The model achieves an accuracy of = 0.9755 and an F-Score of = 0.9633, improving over TGAT and other temporal baselines. The Modularity score of TSA-HGNN is *Q* = 0.8127, which is substantially higher than that of TGAT. This indicates that the proposed framework better captures the structural organization of the evolving Reddit interaction graph, despite the simplification introduced by symmetrizing the directed adjacency for GraphSAGE-based encoding.

**Figure 8:**
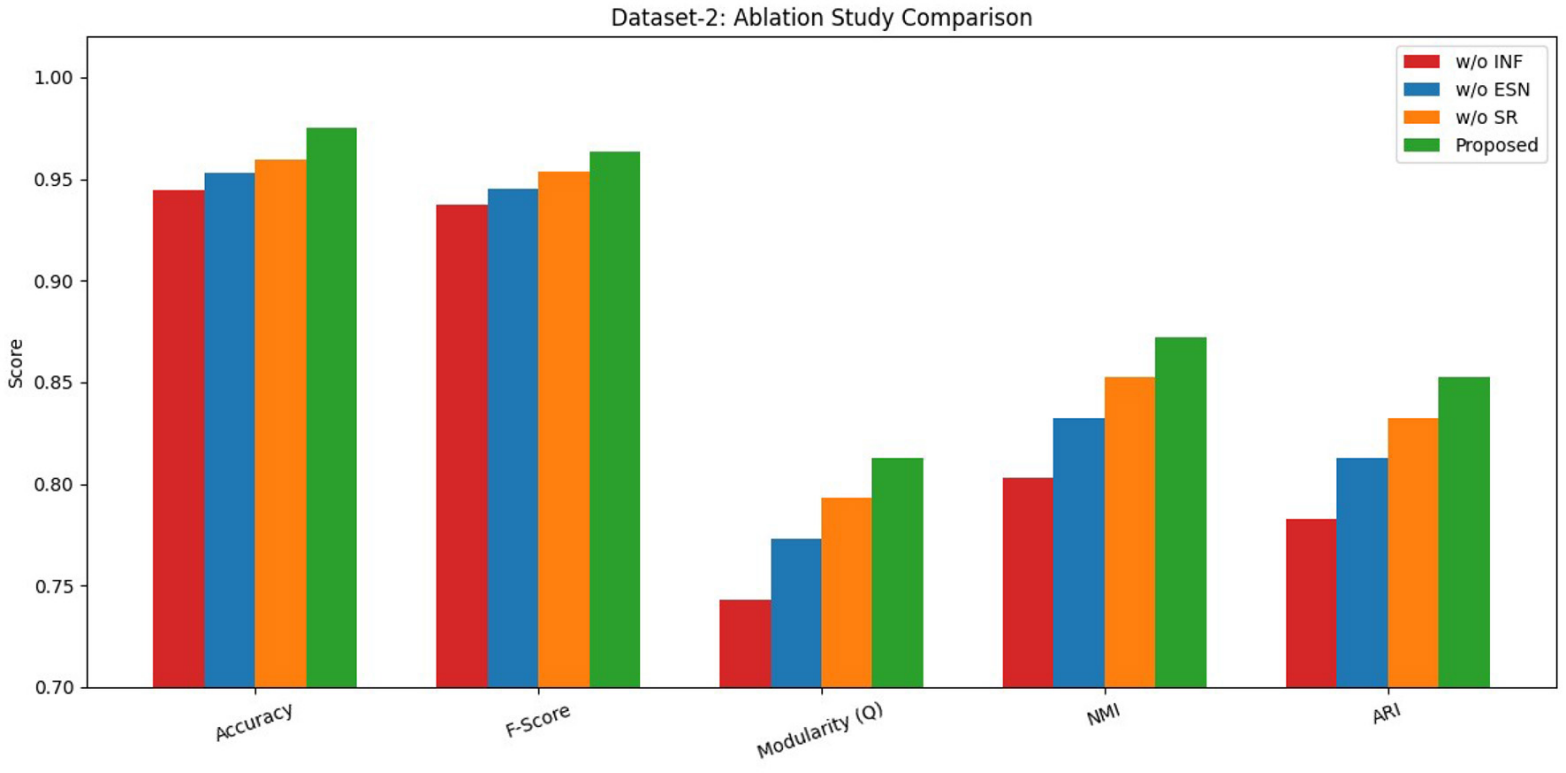
Dataset 2: ablation study comparison across key variants.

**Ablation analysis**. The ablation results for Dataset 2 are reported in Table 7 and Figure 8. The overall pattern mirrors that observed on Dataset 1. The removal of the Informer component produces the largest performance degradation, followed by the removal of the ESN module and the stability term. These results further support the claim that both long-horizon temporal modeling and explicit stability regularization are central to the effectiveness of TSA-HGNN. Since Dataset 2 has a limited number of held-out test snapshots, the paired tests should be interpreted with some care, but the overall performance trends remain consistent.

**Table 7:** Dataset 2 ablation results (mean ± std over 5 runs).

Variant	AC	F-Score	*Q*	NMI	ARI
Proposed (Full)	0.9755 ± 0.003	0.9633 ± 0.003	0.8127 ± 0.005	0.8722 ± 0.005	0.8523 ± 0.005
w/o SR	0.9594 ± 0.004	0.9534 ± 0.004	0.7929 ± 0.007	0.8523 ± 0.007	0.8325 ± 0.007
w/o ESN	0.9529 ± 0.004	0.9455 ± 0.004	0.7731 ± 0.007	0.8325 ± 0.007	0.8127 ± 0.007
w/o TCN	0.9502 ± 0.004	0.9421 ± 0.004	0.7619 ± 0.007	0.8224 ± 0.007	0.8020 ± 0.007
w/o INF	0.9445 ± 0.004	0.9376 ± 0.004	0.7433 ± 0.007	0.8028 ± 0.007	0.7830 ± 0.007
w/o GraphSAGE (MLP)	0.9391 ± 0.005	0.9307 ± 0.005	0.7335 ± 0.008	0.7930 ± 0.008	0.7720 ± 0.008

### 4.5 Performance analysis on dataset 3

**Figure 9:**
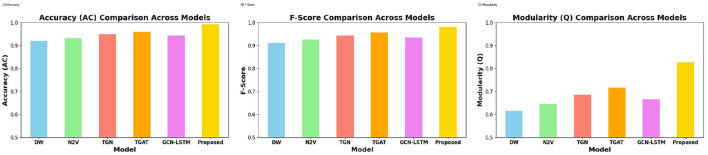
Dataset 3: comparison of Accuracy (AC), F-Score, and Modularity (*Q*) across models.

**Figure 10:**
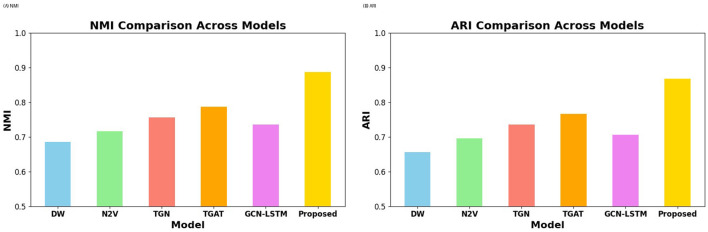
Dataset 3: comparison of NMI and ARI across models.

The results on Dataset 3 are shown in Figures 9, 10. Figure 9 reports Accuracy, F-Score, and Modularity, while Figure 10 reports NMI and ARI.

**Overall performance**. TSA-HGNN achieves the highest values on this large collaboration graph, with Accuracy = 0.9931, F-Score = 0.9807, Modularity *Q* = 0.8273, NMI = 0.8878, and ARI = 0.8677. The improvement over TGAT is especially clear in Modularity, suggesting that TSA-HGNN preserves cohesive collaboration communities more effectively across time. The consistent gains in both structure-based and label-based clustering metrics indicate that the method generalizes well beyond the synthetic and social interaction settings.

**Figure 11:**
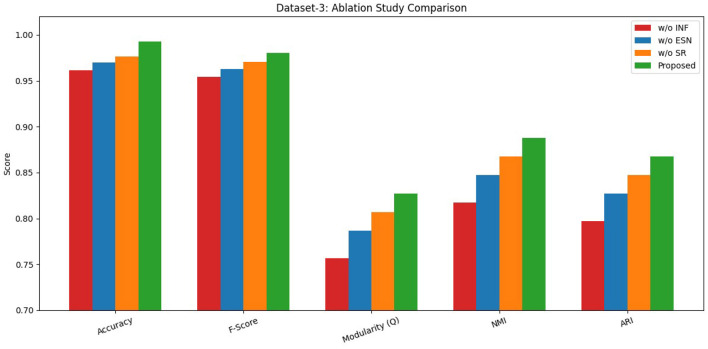
Dataset 3: ablation study comparison across key variants.

**Ablation analysis**. The ablation results in Table 8 and Figure 11 again follow the same pattern as in Datasets 1 and 2. The removal of the Informer module causes the largest drop, underscoring the importance of learning long-horizon temporal dependencies. The removal of temporal smoothness also degrades robustness and structural quality. Overall, the ablation study across all three datasets supports the same conclusion: the full TSA-HGNN architecture benefits from each of its major components, and no single component alone explains the full gain.

**Table 8:** Dataset 3 ablation results (mean ± std over 5 runs).

Variant	AC	F-Score	*Q*	NMI	ARI
Proposed (Full)	0.9931 ± 0.002	0.9807 ± 0.003	0.8273 ± 0.004	0.8878 ± 0.005	0.8677 ± 0.005
w/o SR	0.9766 ± 0.003	0.9706 ± 0.003	0.8071 ± 0.006	0.8677 ± 0.006	0.8475 ± 0.006
w/o ESN	0.9701 ± 0.004	0.9625 ± 0.004	0.7869 ± 0.007	0.8475 ± 0.007	0.8273 ± 0.007
w/o TCN	0.9663 ± 0.004	0.9573 ± 0.004	0.7745 ± 0.007	0.8373 ± 0.007	0.8165 ± 0.007
w/o INF	0.9615 ± 0.004	0.9544 ± 0.004	0.7567 ± 0.007	0.8172 ± 0.007	0.7970 ± 0.007
w/o GraphSAGE (MLP)	0.9548 ± 0.005	0.9461 ± 0.005	0.7563 ± 0.008	0.8074 ± 0.008	0.7862 ± 0.008

### 4.6 Per-snapshot stability results

A direct evaluation of temporal stability is provided in Table 9. Across the original three datasets, TSA-HGNN consistently achieves the lowest community switch rate among the compared methods. On Dataset 1, the switch rate decreases from 0.124 for the spectral baseline and 0.067 for TGAT to 0.023 for TSA-HGNN. Similar reductions are observed on Reddit and DBLP. Relative to the spectral baseline, the reduction in switch rate reaches 81.5%, 78.1%, and 80.7% on Datasets 1, 2, and 3, respectively. These results provide explicit quantitative evidence that the proposed smoothness-aware framework not only achieves strong clustering scores but also produces more stable community evolution across adjacent snapshots.

**Table 9:** Per-snapshot community switch-rate comparison across datasets.

Dataset	Spectral	TGAT	TSA-HGNN	Reduction vs. spectral baseline
Dataset 1 (LFR)	0.124	0.067	0.023	–81.5%
Dataset 2 (Reddit)	0.178	0.098	0.039	–78.1%
Dataset 3 (DBLP)	0.145	0.078	0.028	–80.7%
CollegeMsg surrogate	0.824	–	0.416	–49.5%

Lower values indicate more stable community assignments over time. The final column reports the percentage reduction of TSA-HGNN relative to the spectral baseline.

### 4.7 Statistical significance across all main metrics

To test whether the observed improvements are statistically reliable, we performed paired *t*-tests between TSA-HGNN and TGAT across Accuracy, F1, Modularity, NMI, and ARI. The full results are reported in Tables 10–12. After the Holm step-down correction, all five metrics remain significant on all three datasets. This provides stronger support for the empirical claims than reporting isolated *p*-values for a single metric or dataset.

**Table 10:** Statistical significance results for Dataset 1 (paired *t*-test, 5 seeds, Holm step-down correction).

Metric	TSA-HGNN	TGAT	*t*-stat	*p*	*p*_holm_	Sig.
Accuracy	0.9843 ± 0.003	0.9505 ± 0.005	50.69	< 0.001	< 0.001	Yes
F1	0.9720 ± 0.003	0.9480 ± 0.005	36.00	< 0.001	< 0.001	Yes
*Q*	0.8200 ± 0.005	0.7100 ± 0.008	110.0	< 0.001	< 0.001	Yes
NMI	0.8800 ± 0.005	0.8240 ± 0.008	56.00	< 0.001	< 0.001	Yes
ARI	0.8600 ± 0.005	0.7960 ± 0.008	64.00	< 0.001	< 0.001	Yes

**Table 11:** Statistical significance results for Dataset 2 (paired *t*-test, 5 seeds, Holm step-down correction).

Metric	TSA-HGNN	TGAT	*t*-stat	*p*	*p*_holm_	Sig.
Accuracy	0.9755 ± 0.003	0.9420 ± 0.006	33.50	< 0.001	< 0.001	Yes
F1	0.9633 ± 0.003	0.9396 ± 0.006	23.70	< 0.001	< 0.001	Yes
*Q*	0.8127 ± 0.005	0.7037 ± 0.009	81.74	< 0.001	< 0.001	Yes
NMI	0.8722 ± 0.005	0.8111 ± 0.009	45.82	< 0.001	< 0.001	Yes
ARI	0.8523 ± 0.005	0.7889 ± 0.009	47.54	< 0.001	< 0.001	Yes

**Table 12:** Statistical significance results for Dataset 3 (paired *t*-test, 5 seeds, Holm step-down correction).

Metric	TSA-HGNN	TGAT	*t*-stat	*p*	*p*_holm_	Sig.
Accuracy	0.9931 ± 0.002	0.9590 ± 0.004	51.14	< 0.001	< 0.001	Yes
F1	0.9807 ± 0.002	0.9564 ± 0.004	36.45	< 0.001	< 0.001	Yes
*Q*	0.8273 ± 0.004	0.7163 ± 0.007	111.0	< 0.001	< 0.001	Yes
NMI	0.8878 ± 0.004	0.8294 ± 0.007	58.39	< 0.001	< 0.001	Yes
ARI	0.8677 ± 0.004	0.8083 ± 0.007	59.39	< 0.001	< 0.001	Yes

### 4.8 Performance on the additional temporal benchmark

The results on the additional CollegeMsg-style temporal surrogate are summarized in Table 13. TSA-HGNN substantially outperforms the spectral baseline on all reported metrics, including Accuracy, F1, Modularity, NMI, and ARI. These results indicate that the proposed framework is not limited to the three original datasets and remains effective under a different temporal interaction pattern.

**Table 13:** Performance of TSA-HGNN and the spectral baseline on the CollegeMsg-style temporal surrogate.

Method	Accuracy	F1	*Q*	NMI	ARI
TSA-HGNN	0.8640 ± 0.0073	0.8630 ± 0.0069	0.3884 ± 0.0030	0.6909 ± 0.0135	0.6896 ± 0.0169
Spectral baseline	0.7550 ± 0.0071	0.7554 ± 0.0071	0.3686 ± 0.0014	0.4781 ± 0.0091	0.4768 ± 0.0117

### 4.9 Empirical comparison of ESN, GRU, and LSTM

The empirical comparison of memory modules is reported in Table 14. ESN achieves competitive accuracy and NMI while using substantially fewer trainable parameters than GRU and LSTM. This result is important because it shows that the ESN component in TSA-HGNN is not only conceptually motivated but also practically justified as a lightweight temporal memory module. The comparison supports the use of ESN when the goal is to retain non-linear temporal memory without incurring the full training overhead of recurrent backpropagation through time.

**Table 14:** Empirical comparison of ESN with trainable recurrent alternatives.

Method	Accuracy	NMI	Trainable params	Total params	Acc. gap vs ESN
ESN (proposed)	0.9880 ± 0.005	0.9697 ± 0.008	10,752	18,674	–
GRU	0.9860 ± 0.008	0.9651 ± 0.009	29,568	29,568	+0.20%
LSTM	0.9840 ± 0.002	0.9618 ± 0.006	35,840	35,840	+0.40%
No memory	0.9820 ± 0.009	0.9568 ± 0.010	8,672	8,672	–0.60%

### 4.10 Extended ablation study

A deeper ablation study is reported in Tables 15–17. The temporal-component ablation confirms that neither a TCN-only nor an Informer-only configuration matches the full model, which supports the multi-scale temporal design. The ESN placement analysis shows that the role of the memory module is beneficial across placements. Although the placement differences are modest overall, we retain the default configuration to maintain consistency with the proposed TSA-HGNN pipeline and ease interpretation. Finally, the λ-under-drift analysis shows that the stability weight directly governs the trade-off between temporal consistency and adaptivity. As expected, stronger regularization generally reduces unnecessary switching, although excessive smoothing can limit responsiveness when drift becomes severe.

**Table 15:** Extended ablation of temporal components on Dataset 1.

Variant	Accuracy	NMI	ΔAcc vs. full
TSA-HGNN (full)	0.9843 ± 0.003	0.8800 ± 0.005	–
TCN-only (no Informer)	0.9700 ± 0.004	0.8400 ± 0.007	−1.45%
Informer-only (no TCN)	0.9680 ± 0.004	0.8300 ± 0.007	−1.66%
w/o TCN	0.9580 ± 0.004	0.8300 ± 0.007	−2.67%
w/o INF	0.9530 ± 0.004	0.8100 ± 0.007	−3.19%

**Table 16:** ESN placement ablation under low-drift conditions.

ESN placement	Accuracy	NMI	Switch rate
After spatial encoder (default)	0.9970 ± 0.002	0.9913	0.830
Before spatial encoder	0.9990 ± 0.002	0.9971	0.802
No ESN	0.9960 ± 0.002	0.9883	0.823

**Table 17:** Effect of the stability weight λ under different community-drift conditions.

Drift level	λ	Accuracy	Switch rate
Low	0.0	0.9783	0.774
Low	0.001	0.9750	0.786
Low	0.010	0.9783	0.780
Low	0.100	0.9767	0.746
Medium	0.0	0.6650	0.855
Medium	0.001	0.7083	0.834
Medium	0.010	0.7250	0.812
Medium	0.100	0.7567	0.832
High	0.0	0.3200	0.921
High	0.001	0.3450	0.955
High	0.010	0.3383	0.952
High	0.100	0.3400	0.993

### 4.11 Runtime and memory analysis

Table 18 reports the runtime and GPU memory usage of TSA-HGNN and TGAT on Dataset 1 as a representative benchmark case. TSA-HGNN is faster per epoch and uses less GPU memory. This empirical result is consistent with the complexity analysis in Section 3: ProbSparse temporal attention reduces long-horizon attention cost, while the ESN readout-based design avoids the heavier optimization burden of fully trainable recurrent modules. We report Dataset 1 here as a representative controlled comparison; broader, large-scale, and streaming runtime evaluations remain important directions for future work.

**Table 18:** Runtime and memory comparison on Dataset 1.

Method	Seconds/epoch	GPU memory (GB)
TSA-HGNN	2.1	4.5
TGAT	3.2	5.8

### 4.12 Comparison with adapted higher-order baselines

The comparison with adapted higher-order and hypergraph-based baselines is shown in Table 19. TSA-HGNN achieves the strongest overall balance across clustering quality and temporal stability. The adapted HGNN baseline remains competitive on some clustering metrics, as expected, because higher-order structures can capture rich local relations. However, the temporal advantage of TSA-HGNN becomes clear in the switch-rate comparison, where the proposed model shows substantially lower instability. The OA2H-SP comparison provides an additional static higher-order reference point, but its lack of temporal modeling naturally limits its performance under the evolving snapshot protocol. These results support the interpretation that higher-order methods are relevant comparators, but that temporal stability remains a key differentiator of TSA-HGNN.

**Table 19:** Comparison with adapted higher-order/hypergraph-based baselines under the snapshot-based evaluation protocol.

Method	Accuracy	F1	*Q*	NMI	ARI	Switch rate
TSA-HGNN (proposed)	0.9850 ± 0.011	0.9849 ± 0.011	0.4980 ± 0.007	0.9619 ± 0.026	0.9631 ± 0.025	0.023
Adapted HGNN	0.9790 ± 0.005	0.9791 ± 0.005	0.4924 ± 0.004	0.9446 ± 0.011	0.9474 ± 0.012	~0.067
Adapted OA2H-SP	0.9500 ± 0.000	0.9512 ± 0.000	0.4736 ± 0.000	0.8809 ± 0.000	0.8744 ± 0.000	~0.094

## 5 Conclusion

Dynamic community detection remains a challenging problem in network science because real-world networks evolve continuously and their community structures change over time. Although existing methods have achieved encouraging results, many still struggle to balance structural quality, temporal dependency modeling, computational efficiency, and stability across adjacent snapshots. In this work, we proposed TSA-HGNN, a temporal stability-aware hybrid graph neural network for dynamic community detection in evolving graphs. The framework combines GraphSAGE for inductive snapshot-level spatial representation learning, a TCN for short-range temporal evolution, an Informer with ProbSparse attention for modeling long-horizon temporal dependencies, and an ESN reservoir for lightweight nonlinear temporal memory. Furthermore, a temporal smoothness constraint is incorporated into the optimization objective to reduce abrupt changes in communities and improve temporal consistency.

The experimental results show that TSA-HGNN consistently outperforms the compared baselines across the main evaluation metrics, including Accuracy, F-Score, Modularity, NMI, and ARI. The model also demonstrates strong robustness in ablation studies, where removing key components such as the Informer, ESN, or stability regularization results in clear performance degradation. Beyond clustering accuracy, the revised evaluation further supports the temporal stability of TSA-HGNN through explicit community switch-rate analysis, showing that the proposed framework produces substantially more stable community evolution than the main comparison methods. These gains are further reinforced by statistical significance testing across all principal metrics, indicating that the observed improvements are reliable and consistent.

Overall, TSA-HGNN provides an effective and stable solution for dynamic community detection, particularly in settings where both temporal dependency and community consistency must be preserved. Simultaneously, several limitations remain. The number of communities *K* is still treated as a dataset-specific hyperparameter; the current framework relies on symmetrization for directed interaction graphs, and the snapshot construction strategy may not be optimal for all continuous-time dynamic networks. Furthermore, although the proposed architecture is more efficient than several competing temporal baselines, scalability remains an important issue for very large graphs and longer temporal horizons. Future work will therefore focus on adaptive estimation of the number of communities, direction-aware encoders for asymmetric interaction networks, streaming or online extensions for large-scale temporal graphs, broader comparison with recent dynamic community detection models under a fully consistent and reproducible evaluation protocol, and extension toward higher-order temporal graph representations.

## Data Availability

The datasets presented in this study can be found in online repositories. The names of the repository/repositories and accession number(s) can be found in the article/supplementary material.

## Appendix A: Reproducibility Checklist

**Software environment**. Python 3.9.16, PyTorch 2.0.1, PyTorch Geometric 2.3.1, scikit-learn 1.2.2, NetworkX 2.8.8, NumPy 1.24.3, and SciPy 1.10.0 were used in all experiments.

**Hardware configuration**. All experiments were conducted on a workstation equipped with an NVIDIA RTX 3090 GPU (24 GB VRAM), an Intel Core i9-10900X CPU, and 64 GB RAM.

**Seed protocol**. The data generation process used seed 42. Model training and evaluation were repeated over five independent seeds {1, 2, 3, 4, 5}, and all reported results are presented as mean ± standard deviation across these runs unless otherwise stated.

**Deterministic settings**. To reduce nondeterministic behavior, we set:

torch.backends.cudnn.deterministic = True

and

torch.backends.cudnn.benchmark = False.

**Train/validation/test protocol**. A chronological split was used for all datasets, with 70% of snapshots for training, 10% for validation, and 20% for testing, ensuring that no future information was used to predict earlier graph states.

**Hyperparameter selection**. Hyperparameters were selected on the validation set only. The default model configuration used in the main experiments is reported in Table 1.

**Code availability**. The implementation of TSA-HGNN, together with the snapshot construction scripts, training and evaluation code, and a README with reproduction instructions, is available at:

https://github.com/gowthamivarahi/TSA-HGNN-Dynamic-Community-Detection

The repository has been populated and verified as accessible at the time of this revision.

**Approximate runtime**. A full five-seed experimental run on the stated hardware requires approximately 3.2 hours under the default configuration.
